# Performance indicators for public mental healthcare: a systematic international inventory

**DOI:** 10.1186/1471-2458-12-214

**Published:** 2012-03-20

**Authors:** Steve Lauriks, Marcel CA Buster, Matty AS de Wit, Onyebuchi A Arah, Niek S Klazinga

**Affiliations:** 1 Department of Epidemiology, Documentation and Health Promotion EDG, Municipal Health Service Amsterdam, Nieuwe Achtergracht 100, 1018 WT Amsterdam, The Netherlands; 2 Department of Public Health, Academic Medical Center-University of Amsterdam, Meibergdreef 2, 1105 AZ Amsterdam, The Netherlands; 3 Department of Epidemiology, UCLA School of Public Health, 650 Charles E. Young Drive South, Los Angeles, CA 90095-1772, USA

## Abstract

**Background:**

The development and use of performance indicators (PI) in the field of public mental health care (PMHC) has increased rapidly in the last decade. To gain insight in the current state of PI for PMHC in nations and regions around the world, we conducted a structured review of publications in scientific peer-reviewed journals supplemented by a systematic inventory of PI published in policy documents by (non-) governmental organizations.

**Methods:**

Publications on PI for PMHC were identified through database- and internet searches. Final selection was based on review of the full content of the publications. Publications were ordered by nation or region and chronologically. Individual PI were classified by development method, assessment level, care domain, performance dimension, diagnostic focus, and data source. Finally, the evidence on feasibility, data reliability, and content-, criterion-, and construct validity of the PI was evaluated.

**Results:**

A total of 106 publications were included in the sample. The majority of the publications (n = 65) were peer-reviewed journal articles and 66 publications specifically dealt with performance of PMHC in the United States. The objectives of performance measurement vary widely from internal quality improvement to increasing transparency and accountability. The characteristics of 1480 unique PI were assessed. The majority of PI is based on stakeholder opinion, assesses care processes, is not specific to any diagnostic group, and utilizes administrative data sources. The targeted quality dimensions varied widely across and within nations depending on local professional or political definitions and interests. For all PI some evidence for the content validity and feasibility has been established. Data reliability, criterion- and construct validity have rarely been assessed. Only 18 publications on criterion validity were included. These show significant associations in the expected direction on the majority of PI, but mixed results on a noteworthy number of others.

**Conclusions:**

PI have been developed for a broad range of care levels, domains, and quality dimensions of PMHC. To ensure their usefulness for the measurement of PMHC performance and advancement of transparency, accountability and quality improvement in PMHC, future research should focus on assessment of the psychometric properties of PI.

## Background

Public mental healthcare (PMHC) systems are responsible for the protection of health and wellbeing of a community, and the provision of essential human services to address these public health issues [1,2]. The PMHC-system operates on three distinct levels of intervention. At a population-level, PMHC-services promote wellbeing of the total population within a catchment area. At a risk group-level, PMHC-services are concerned with the prevention of psychosocial deterioration in specific subgroups subject to risk-factors such as long-term unemployment, social isolation, and psychiatric disorders. Finally, at an individual care-level, PMHC-services provide care and support for individuals with severe and complex psychosocial problems who are characterized either by not actively seeking help for their psychiatric or psychosocial problems, or by not having their health needs met by private (regular) health care services [3]. However, a service developed or initially financed with public means, as a reaction to an identified hiatus in the private health care system, may eventually be incorporated in the private health care system. The dynamics of this relation between the public and private mental health care systems are determined locally by variations in the population, type and number of health care providers, and the available public means. Thus, the specific services provided by the PMHC system at any moment in time differs between nations, regions, or even municipalities.

At the individual care-level, four specific functions of PMHC can be identified [4]. 1) guided referral, which includes signaling and reporting (multi-) problem situations, making contact with the client, screening to clarify care-needs, and executing a plan to guide the client to care, 2) coordination and management of multi-dimensional care provided to persons that present with complex clinical conditions, ensuring cooperation and information-exchange between providers (e.g. mental health-, addiction-, housing- and social services), 3) develop and provide treatment that is not provided by private healthcare organizations, often by funding private healthcare organizations to provide services for specific conditions (e.g. early psychosis intervention services, or methadone maintenance services), and 4) monitoring trends in the target group.

Accountability for services and supports delivered, and funding received, is becoming a key component in the public mental health system. As part of a health system, each organization is not only accountable for their own services, but has some responsibility for the functioning of the system as a whole as well [5]. International healthcare organizations, as well as national and regional policymakers are developing performance indicators (PI) to measure and benchmark the performance of health care systems as a precondition for evidence-based health policy reforms. [e.g. [6-11]]. Many organizations have initiated the development and implementation of quality assessment strategies in PMHC. However, a detailed overview of PI for PMHC is lacking.

To provide an overview of the current state of PI for PMHC we conducted a structured review of publications in scientific peer-reviewed journals supplemented by a systematic inventory of PI published in policy documents and reports by (non-) governmental organizations (so-called 'grey literature'). First, the different initiatives on performance measurement in PMHC-systems and services were explored. Second, the unique PI were categorized according to their characteristics including domain of care (i.e. structure, process or outcome), dimension of quality (e.g. effectiveness, continuity, and accessibility), and method of development (e.g. expert opinion, or application of existing instruments). Finally, we assessed the evidence on the reliability and validity of these performance measures as indicators of quality for public mental healthcare.

## Methods

Publications reporting on PI for PMHC were identified through database- and internet searches. Ovid Medline, PsychInfo, CINAHL and Google (scholar) searches were conducted using any one of the following terms and/or mesh headings, on (aspects of) PMHC: 'mental health system', 'public health system', 'mental health services','public health services', 'mental health care', 'public health care', 'state medicine', 'mental disorders', 'addiction', 'substance abuse', 'homeless', and 'domestic violence'; combined with any one of the following terms/mesh headings on performance measurement: 'quality indicator','quality measure', performance indicator', 'performance measure', and 'benchmarking'.

Database searches were limited to literature published in the period between 1948 and 2010; Google search was conducted in October 2009. Included websites were revisited in February 2011 to check for updates. Publications had to be in the English or Dutch language to be included. Studies, reports and websites were included for further review if a focus on quality measurement of healthcare services related to PMHC became apparent form title, header, or keywords. Abstracts and executive summaries were reviewed to exclude publications on somatic care; elderly care; children's healthcare; and healthcare education. Final selection was based on review of the full content, excluding publications that did not specify the measures applied to assess health care performance. Reference lists of the included publications were reviewed to assure all relevant publications were included in the final sample. Generally, all publically funded services aimed at the preservation, maintenance, improvement of the mental and social health of an adult population, risk-group or individual were considered part of the PMHC system. However, publications on PI designed for private mental health care were included when these PI were applied, or referred to, in publications on PMHC quality assessment.

Included publications were ordered by nation or region. Publications from the same nation were ordered chronologically. Subsequently, we assessed the objective of the publication, the designation of the proposed PI (-set) or quality framework, and the purpose of the proposed PI (-set) or quality framework.

The individual PI were then classified by the following characteristics: a) method of development; b) level of assessment; c) domains of care as proposed by Donabedian [12]; d) dimensions of performance; e) focus on specific diagnosis or conditions; and f) data source. In some cases, the care domain, and/or dimension of performance were not explicitly reported in the publication. The missing domain or dimension was then specified by the author based on: 1) commonly used dimensions in that region as described by Arah et al. [13]; 2) purpose and perspective of the quality framework; and 3) similar PI from other publications for which a domain and/or dimension was specified.

Finally, evidence on the feasibility, data reliability and validity of the included PI was reviewed. Feasibility of PI refers to the possibility that an indicator can be implemented in the PMHC-system or service, given the current information-infrastructure and support by the field. Data reliability refers to the accuracy and completeness of data, given the intended purposes for use [14]. Three forms of validity are distinguished: a) Content-related validity, which refers to evidence that an indicator covers important aspects of the quality of PMHC. b) Criterion-related validity, which refers to evidence that an indicator is related to some external criterion that is observable, measurable, and taken as valid on its face. c) Construct-related validity, which refers to evidence that an indicator measures the theoretical construct of quality and/or performance of PMHC [15,16].

## Results

### Publications on PMHC quality measurement

The library-database and internet search resulted in 3193 publications in English- and Dutch- language peer-reviewed journals and websites from governmental as well as nongovernmental organizations. Further selection based on title- and keyword criteria resulted in the inclusion of approximately 480 publications. After reviewing the abstracts, 152 publications on quality measurement in adult (public) mental health care were included. Final selection based on full publication content resulted in the exclusion of another 46 publications that did not explicitly specify the measures applied to assess health care performance, leaving 106 publications to be included in the final sample.

Table 1 shows the included publications structured by nation/region and date of (first) publication.

**Table 1:** Publications and PMHC quality measurement initiatives per nation/region

Nation/region	Author/organization (year)	Objective of publication/study	PI/sets/frameworks	Purpose of PI/set/framework
*USA*				
	Simpson & Lloyd (1979) [17]	Cohort study relating client perception of program performance to outcomes	Client evaluations of drug abuse treatment in relation to follow-up outcomes	Assess drug treatment effectiveness
	Koran & Meinhardt (1984) [18]	Assessment of validity of County Need Index	Social indicators in statewide mental health planning: lessons from California	Promote equity in the distribution of mental health funds
	National Committee for Quality Assurance (since 1993) [19]	PI development, assessment of usefulness and feasibility, and implementation	Health Plan/Employer Data Information Set (HEDIS)	Help employers to evaluate and compare performance among HMOs and other health plans
	McLellan et al. (1994) [20]	Exploration of patient and treatment factors in outcomes	Similarity of outcome predictors across opiate, cocaine, alcohol treatments; role of treatment services	Evaluate effectiveness of substance abuse treatment in reducing substance use, and improving social adjustment.
	Mental Health Statistics Improvement Program (1996) [21]	PI development, review of quality measurement performance initiatives	MHSIP Consumer-oriented Mental health Report Card	Capture and reflect important characteristics of mental health service delivery
	Srebnik et al. (1997) [22]	PI development based on literature review and stakeholder-opinion, assessment of PI validity	Outcome indicators for monitoring the quality of public mental health care	Assess the quality of public mental health care by consumers and providers
	Lyons et al. (1997) [23]	Determine whether readmissions can service as a PI for an inpatient psychiatric service	Predicting readmission to psychiatric hospital in a managed care environment: implications for quality indicators	Provide program managers, third-party payers, and policy makers with information regarding the functioning of health services
	Baker (1998) [24]	PI development and presentation of method of quality monitoring	A PI spreadsheet for physicians in community mental health centers	Demonstrate progress in meeting objectives and implementing strategies for mental health care to legislators and stakeholders
	Carpinello et al. (1998) [25]	Explore development, implementation, and early results of using a comprehensive performance management system	Managing the performance of mental health managed care: an example from New York State's Prepaid Mental Health Plan	Reflect the concerns of multiple stakeholders and form a foundation for continuous quality improvement activities and information-reporting products
	Pandiani et al. (1998) [26]	PI development and assessment of PI sensitivity and usefulness	Using incarceration rates to measure mental health program performance	Provide program administrators with standardized information of program performance in the area of mental health care
	Rosenheck & Cicchetti (1998) [27]	PI development and implementation	Mental health program report card for public sector programs	Tool in improvement of service delivery, mental health system performance, and accountability
	Macias et al. (1999) [28]	Assess the worth of mental health certification as a core component of state and regional performance contracting	The value of program certification for performance contracting	Assess the quality and fidelity of 'clubhouse' psychiatric rehabilitation programs
	Baker (1999) [29]	Description of management process for financial and clinical PI	PI for physicians in community mental health centers	Report clinical and financial performance to payers of mental health services
	Druss et al. (1999) [30]	Examine the association between consumer satisfaction- and administrative measures at an individual and a hospital level	Patient satisfaction and administrative measures as indicators of the quality of mental health care	Provide providers, purchasers and consumers with understandable and measurable information on the quality of health care
	Department of Health and Human Services (2000) [31]	Present a comprehensive, nationwide health promotion and disease prevention agenda.	Healthy People 2010--Understanding and improving health	Guiding instrument for addressing health issues, reversing unfavorable trends, and expanding past achievements in health
	Huff (2000) [32]	Assess the association between measures of post-admission outpatient utilization and readmission	Outpatient utilization patterns and quality outcomes after first acute ePIode of mental health hospitalization	Provide state, patient advocates and service providers with information to ensure outpatient quality of care
	McCorry et al. (2000) [33]	PI development and adoption of core set of PI by health plans, private employers, public payers, and accrediting associations	The Washington Circle Group core set of PI for alcohol- and other drug services for public- and private sector health plans	Promote quality and accountability in the delivery and management of AOD abuse services by public and private organized systems of care
	Vermont's Mental Health Performance Indicator Project Multi-stakeholder Advisory Group (2000) [34]	Recommendations for PI to be included in a publicly available mental health report card	Indicators of mental health program performance	Development of a data based culture of learning about the system of care
	National Association of State Mental Health Program Directors (2000) [35]	Provide a guide and a framework for the implementation of PI in mental health systems	The NASMHPD framework of mental health PI	Address the need for a standardized methodology for evaluating the impact of services provide through the public mental health system
	Siegel et al. (2000) [36]	Framework development and selection of performance measures	PI of cultural competency in mental health organizations	Assess the cultural competency of mental health systems
	American college of Mental Health Administration (2001) [37]	PI development, reaching consensus between five national accreditation organizations on quality assessment and measurement	A proposed consensus set of PI for behavioral health	Advance the partnership between consumers, purchasers, providers and others in quality measurement and improvement
	Young et al. (2001) [38]	Estimate the rate of appropriate treatment, and the effect of insurance, provider type and individual characteristics on receipt of appropriate care	Survey to assess quality of care for depressive and anxiety disorders in the US	Evaluate mental health care quality on a national basis
	California Department of Mental Health (2001) [39]	PI development and identify areas that require special study of feasibility of measures	PI for California's public mental health system	Provide information needed to continuously improve the care provided in California's public mental health system
	Eisen et al. (2001) [40]	Provide data that could be used to develop recommendations for an improved consumer survey	Toward a national consumer survey: evaluation of the CABHS and MHSIP instruments	Assess quality of behavioral health from consumer perspective
	Chinman et al. (2002) [41]	Illustrate the utility of a continuous evaluation system in promoting improvements in a mental health treatment system	The Connecticut Mental Health Center patient profile project: application of a service need index	Defining the characteristics of the patient population to guide management decisions in caseload distribution and service development
	Davis & Lowell (2002a, b) [42,43]	Demonstrate the value of proper proportions of resources	a. Expenditure on, and b. fiscal structure of mental health care systems and its relationship to suicide rate	Calculate the optimum distribution of community/state psychiatric hospital beds, and cost per capita for mental health care to minimize suicide rate
	Dausey et al. (2002) [44]	Examine the relationship between preadmission care and length of inpatient stay, access to aftercare, and re-hospitalization	Preadmission care as a new mental health PI	Assess the quality, continuity, and intensity of care
	Minnesota Department of Human Services (2002) [45]	Inform counties and providers of the implementation of PI	PI measures for Adult Rule 79 mental health case management	Report on outcomes from the adult mental health system to comply with state's federal mental health block grant application
	Hermann et al. (2002) [46]	Assess utility and applicability of process measures for schizophrenia care	National inventory of measures of clinical processes proposed or used in the U.S.	Assess quality of care for schizophrenia
	Pandiani et al. (2002) [47]	Provide a methodological outline for measuring access and identify and discuss a set of decision points in the project	Measuring access to mental health care: a multi-indicator approach to program evaluation	Assess access to publicly funded systems focusing on both general and special populations
	Druss et al. (2002) [48]	Asses the relation between mental health care quality measures and measures of general care quality	HEDIS 2000 mental health care PI	Provide purchasers a report card for rating and selecting health plans
	CDC--National Public Health Performance Standards Program, (NPHPSP; 2002) [49]	Present instruments for assessment of local and state public health systems	Local and State public health system performance assessment instruments & Local public health governance performance assessment instrument	To improve the practice of public health by comprehensive performance measurement tools keyed to the 10 Essential Services of Public Health
	Beaulieu & Scutchfield (2002) [50]	Assess the face and content validity of NPHPSP instrument	Local Public Health System Performance Assessment Instrument	Ensure the delivery of public health services and support a process of quality improvement
	Beaulieu et al. (2003) [51]	Assess the content and criterion validity of NPHPSP instruments	Local and State Public Health System Performance Assessment instruments	Measure performance of the local and state public health system
	Trutko & Barnow (2003) [52]	Explore feasibility of developing a core set of PI measures for DHHS programs that focus on homelessness	Core PI for homeless-serving programs administered by the US DHHS	Facilitate documentation and analysis of the effectiveness of program interventions
	The Urban Institute (2003) [53]	Describe lessons learned from PI development experiment and provide suggestions for other communities	Community-wide outcome indicators for specific services	Balance outcome-reporting requirements of funders for accountability and providers for improvement of services
	Greenberg & Rosenheck (2003) [54]	Examine the association of continuity of care with factors (not) under managerial control	Managerial and environmental factors in the continuity of mental health care across institutions	Assess the quality of outpatient care for persons with severe mental illness
	Owen et al. (2003) [55]	Examine meaningfulness and validity of PI and automated data elements	Mental health QUERI initiative: expert ratings of criteria to assess performance for major depressive disorder and schizophrenia	Provide clinicians, managers, quality improvement specialists and researchers in the Veterans Health Administration with useful data on clinical practice guidelines compliance
	Siegel et al. (2003) [56]	Benchmarking selected performance measures	PI of cultural competency in mental health organizations	Assess organizational progress in attaining cultural competency (CC) and to provide specific steps for implementing facets of CC.
	Solberg et al. (2003) [57]	Understand the process, outcomes and patient satisfaction of primary care patients diagnosed with depression	Process, outcomes and satisfaction in primary care for patients with depression	Identify quality gaps and serve as a baseline for quality improvements in health plan depression care
	Center for Mental Health Services (CMHS), Substance Abuse and Mental Health Service Administration (SAMHSA), DHHS (2003) [58]	Report on 16-state indicator pilot project focused on assessment, refinement an pilot testing comparable mental health performance indicators	PI adopted from the NASMHPD Framework of Performance Indicators reflecting much of the MHSIP Report Card	Report mental health system performance comparably across states for national reporting, and facilitate planning, policy formulation and decision making at the state level.
	Edlund et al. (2003) [59]	Validate the technical quality-satisfaction relationship and examine the effects of selection bias among patients with depressive and anxiety disorders	Satisfaction measures as a reflection of technical quality of mental health care	Provide health care plan and provider quality information to insurers, providers, and researchers for improvement of quality of care for common mental disorders
	Virginia Department of Mental Health, Mental Retardation and Substance Abuse Services (2003) [60]	PI implementation and report on outcomes	Virginia's performance outcomes measurement system (POMS)	Provide public mental health authorities with information on consumer outcomes and provider performance to contain costs, improve quality and provide greater accountability
	Blank et al. (2004) [61]	Assess efficiency of a selection of POMS indicators and develop recommendations for improving POMS	Virginia's POMS	Continuously improve the quality of services and increase accountability for taxpayer dollars
	Charbonneau et al. (2004) [62]	Explore the relationship of process measures with subsequent overall hospitalizations	Guideline-based depression process measures	Estimate healthcare quality and quantify its benefits
	Stein et al. (2004) [63]	Evaluate the process and quality of care and examine patient characteristics that potentially determine quality	Quality of care for patients with a broad array of anxiety disorders	Assess the quality of care received in primary care settings for efforts at quality improvement
	Druss et al. (2004) [64]	Assess relation between mental health care volume and quality	HEDIS 2000 mental health care PI	Reflect the capacity to treat specialized conditions and as proxy for clinician volume
	McGuire & Rosenheck (2004) [65]	Examine the relation between incarceration history and baseline psychosocial problems service utilization, and outcomes of care	Criminal history as a prognostic indicator in the treatment of homeless people with severe mental illness	Provide clinicians and administrators with information on treatment prospects of former inmates
	Leff et al. (2004) [66]	Investigate the relationship between service fit and mortality as a step towards understanding the general relationship between service quality and outcomes	Service quality as measured by service fit vs. mortality among public mental health system service recipients	Assess and compare programs and systems, the extent to which an intervention has been implemented in program evaluations, an service need in program and resource allocation planning
	Valenstein et al. (2004) [67]	Examine providers' views of quality monitoring processes and patient, provider and organizational factors that might be associated with more positive views	PI drawn from sets maintained and implemented by various national organizations	Provide mental health care providers with feedback about their performance
	Mental health recovery: What helps and what hinders? A National Research Project for the Development of Recovery Facilitating System Performance Indicators (2004) [68]	PI development, and assessment of usability and implementation	Recovery oriented system indicators (ROSI)	Facilitate mental health recovery, and bridge the gap between the principles of recovery and self-help and application of these principles in everyday work of staff and service systems
	Hermann et al. (2004) [69]	PI selection and assessment of PI meaningfulness and feasibility	Core set of PI for mental and substance-related care	Ensure that systems and providers focus on clinically important processes with known variations in quality of care
	Rost et al. (2005) [70]	Explore relation between administrative PI and absenteeism	Relationship of depression treatment PI to employee absenteeism	Provide employers with evidence of the value of the healthcare they purchase.
	Mental Health Statistics Improvement Program (2005) [71]	PI development and present toolkit for methodology, implementation and uses	MHSIP Quality Report (MQR)	Reflect key concerns in mental health systems or organizations performance
	Washington State Department of Social and Health Services--Mental Health Division (2005) [72]	PI implementation and report on PI information	State-wide publicly funded mental health PI	Help system managers and payers understand trends in services delivery systems and change across time
	New York Office of Mental Health (2005) [73]	PI development and implementation	2005-2009 Statewide comprehensive plan for mental health services	Provide a conceptual framework for performance measurement and improvement
	Garnick et al. (2006) [74]	Examine different types of PI, how they fit within the continuum of care, and the types of data that can be used to arrive at these measures	PI for alcohol and other drug services	Evaluate how well practitioners' actions conform to guidelines, review criteria or standards to improve access, and quality of treatment
	Hermann et al. (2006) [75]	Develop statistical benchmarks for quality measures of mental health and substance-related care	Selected measures from core set of PI for mental and substance-related care	Assess quality of care for Medicaid beneficiaries to inform quality improvement
	Mental health recovery: What helps and what hinders? A National Research Project for the Development of Recovery Facilitating System Performance Indicators (2006) [76]	Refinement of self-report survey and administrative profile PI based on feedback from stakeholders	Recovery oriented system indicators (ROSI)	Measure critical elements and processes of recovery facilitating mental health programs and delivery systems
	Busch et al. (2007) [77,78]	PI development informed by APA guidelines for the treatment of bipolar disorder	Quality of care for bipolar I disorder	Assess quality of medication and psychotherapy treatment
	Center for Quality Assessment and Improvement in Mental Health (2007) [79]	PI development using an adaptation of the RAND appropriateness method, and assess reliability	Standards for bipolar excellence (STABLE) PI	Advance the quality of care for by supporting improved recognition and promoting evidence-based management
	CDC--National Public Health Performance Standards Program (NPHPSP; 2007) [80]	Present the revised instruments for assessment of local and state public health systems	Version 2.0 of the Local and State public health system performance assessment instruments and Local public health governance performance assessment instrument	Provide users with information to identify strengths and weaknesses of the public health system to determine opportunities for improvement
	Virginia Department of Mental Health, Mental Retardation and Substance Abuse services (2008) [81]	PI implementation and report on achieved goals	2008 mental health block grant implementation report PI	Monitor the implementation and transformation of a recovery-oriented system

*Canada*				
	Canadian Institute for Health Information (CIHI; 2001) [82]	PI development, assessment of feasibility & usefulness	The Roadmap Initiative--Mental health and Addiction Services Roadmap Project. Phase 1 Indicators	Maintain and improve Canada's health system
	Federal/Provincial/Territorial Advisory Network on Mental Health (2001) [83]	PI development	PI for Mental health Services and Supports--A Resource Kit	Facilitate ongoing accountability and evaluation of mental health services and supports
	Ontario Ministry of Health and Long-term Care (2003) [84]	PI development and mechanisms for implementation	Mental Health Accountability Framework	Increasing health system accountability to ensure services are as effective and efficient as possible
	Addington et al. (2005) [85]	PI selection based on literature review and consensus procedure	PI for early psychosis treatment services	Evaluate quality, and assist providers in improving quality of health care

*Australia*				
	NMHWG Information Strategy committee Performance Indicator drafting group (2005) [86]	Development conceptual framework of performance & PI	Key PI for Australian public mental health services	Improve public sector mental health service quality
	Meehan et al. (2007) [87]	Assessment of feasibility & usefulness of benchmarking mental health services	Input, process, output and outcome PI for inpatient mental health services	Benchmarking public sector mental health service organizations

*United Kingdom*				
	Jenkins (1990) [88]	PI development	A system of outcome PI for mental health care.	Ensure that clinicians district health authorities and directors of public health can monitor and evaluate mental health care
	National Health Service (1999a, b) [89,90]	Framework and PI development	A National Service Framework for Mental Health; A New Approach To Social Services Performance	Help drive up quality and remove the wide and unacceptable variations in provision.
	Shipley et al. (2000) [91]	PI development and validity assessment	Patient satisfaction: a valid index of quality of care in a psychiatric service	Provide PMHC planners with an independent yardstick for mental health services and determine population mental health
	Audit Commission (2001) [92]	PI development and application	Library of Local Authority PI	Accountability and benchmarking of local authorities by national government
	Jones (2001) [93]	Review of pre-existing PI	Hospital care pathways for patients with schizophrenia	Clarify terms and concepts in schizophrenia care process
	Shield et al. (2003) [94]	PI development	PI for primary care mental health services	Facilitating quality improvement and show variations in care
	Commission for Health Improvement (2003) [95]	PI development and implementation	Mental health trust balanced scorecard indicators	Improve care provided by mental health trusts and promote transparency in PMHC
	Department of Health (2004) [96]	PI development	National Standards, Local Action--health and social care standards and planning framework	Set out the framework for all NHS organizations and social service authorities to use in planning over the next financial three years
	NHS Health Scotland (2007) [11]	PI development based on current data, policy, evidence, and expert-opinion	Core set of national, sustainable mental health indicators for adults in Scotland	Determine whether mental health is improving and track progress
*UK (cont.)*	Care Services Improvement Partnership (2007) [97]	PI development	Outcome indicators framework for mental health day services	Help commissioners and providers to monitor, evaluate, and measure the effectiveness of day services adults with mental health problems
	Healthcare Commission (2007) [98]	PI development	The Better Metrics Project	Provide a common set of requirements to ensure safe and acceptable quality health provision, and provide a framework for continuous improvement
	Department of Communities and Local Government (2007) [99]	PI development and application	The National Indicator Set (NIS) in Comprehensive Area Assessment (CAA)	Performance management of local government by central government
	Association of Public Health Observatories (2007) [100]	Present data on the factors which give rise to poor mental health, mental health status of populations, provision of interventions, service user experience and traditional outcomes	Indications of public health in the English Regions: Mental Health	Provide a resource for regional public health directors, PCT and CSIP directors in making decisions, holding to account those responsible for the delivery and improving mental health of the population.
	Wilkinson et al. (2008) [101]	Report on the construction of a set of indicators for mental health and the publication of a report for England's Chief Medical Officer	Indications of public health in the English Regions: Mental Health	Initiating public health action to improve health at a regional level in England
	London Health Observatory (2008) [102]	PI development and implementation	Mental health and wellbeing scorecard	Support primary care trusts in monitoring delivery of national health improvement objectives, and improvement of mental health and wellbeing
	Care Services Improvement Partnership (2009) [103]	Broaden initial framework to provide for application in mental health services more widely	Outcome indicators framework for mental health services	Ensure the effectiveness and impact of redesigned and refocused services
	Association of Public Health Observatories (2009) [104]	PI development, application of pre-existing PI, operationalization of issues, targets and recommendations in policies	Indications of public health in the English regions: Drug Use	Present information on the relative positions of regions on major health policy areas, highlighting differences, to stimulate practitioners to take action to improve health

*Spain*				
	Gispert et al. (1998) [105]	PI development, assessment of feasibility	Mental health expectancy: a global indicator of population mental health	Reflect the impact that disability due to mental disorders has on population health

*Germany*				
	Kunze & Priebe (1998) [106]	Development of quality assessment tool	Assessing the quality of psychiatric hospital care: a German approach.	Assessment of quality of care after political reforms to help promote quality.
	Bramesfeld et al. (2007) [107]	Implementation of quality assessment tool	Evaluating inpatient and outpatient care in Germany with the WHO responsiveness concept	Evaluate performance of mental health care services to improve responsiveness

*The Netherlands*				
	Roeg et al. (2005) [108]	Development of disease-specific concept of quality	Conceptual framework of quality for assertive outreach programs for severely impaired substance abuses	Improve understanding of the relationship between specific program features and effectiveness
	Nabitz et al. (2005) [109]	Development of disease-specific concept of quality	A quality framework for addiction treatment programs	Clarify the concept of quality for addiction treatment programs
	Nieuwenhuijsen et al. (2005) [110]	PI development & validity assessment	PI for rehabilitation of workers with mental health problem	Assessment of occupational health care to improve the quality of care
	Wierdsma et al. (2006) [111]	Application & risk adjustment of PI	Utilization indicators for quality of involuntary admission mental health care	Assess criteria for involuntary admission to inpatient mental health care
*The Netherlands (cont.)*	Steering Committee--Transparency Mental Healthcare (2007) [112]	Improvement of existing PI and PI development	Basic Set of PI for Mental Health Care and Addiction Care services	Promoting transparency and publication of quality information by mental health and addiction service providers

*Italy*				
	Bollini et al. (2008) [113]	PI development, operationalization of (PORT) guidelines	Indicators of conformance with guidelines of schizophrenia treatment in mental health services	Monitor the conformance of care with recommend practices and identify areas in need of improvement

*South Africa*				
	Lund & Fisher (2003) [114]	PI development and assessment of PI usefulness	Community/hospital indicators in South African public sector mental health services	Assess the implementation of policy objectives over time

*Singapore*				
	Chong et al. (2006) [115]	Application of pre-existing PI and operationalization of guidelines	Assessment of the quality of care for patients with first- episode psychosis	Assess adherence to guidelines in an early psychosis intervention program

*International*				
	National Research and Development Centre for Welfare and Health (STAKES)--EC Health Monitoring Programme (2002) [8]	PI development and assessment of feasibility and usability	A set of mental health indicators for European Union	Contribute to the establishment of a community monitoring system
	Organisation for Economic Cooperation and Development (OECD; 2004) [10]	PI selection and assessment of utility	Indicators for the quality of mental health care at the health system level in OECD countries	Improve organization and management of care to allow countries to spend their health care dollars more wisely
	World Health Organization (2005) [116]	PI development, operationalization of recommendations, assessment of usefulness	Assessment Instrument for Mental Health Systems (WHO-AIMS) version 2.2	Collect essential information on the mental health system of a country or region to improve mental health systems
	Saxena et al. (2006) [117]	Describe and compare 4 existing high-income country public mental health indicator schemes	Healthy People 2010; Mental Health Report Card (MHSIP); Commission for Health Improvement Indicators (CHI); European community Health Indicators (ECHI)	Contribute to the development of relevant policies and plans
	Hermann et al. (2006) [118]	Report on methods employed to reach consensus on the OECD mental health care indicators	Indicators for the quality of mental health care at the health system level in OECD countries	Facilitate improvement within organizations, provide oversight of quality by public agencies and private payers, and provide insight into what levels of performance are feasible
	OECD (2008) [119]	Provide overview of present mental health care information systems to assess feasibility of performance indicators	Indicators for the quality of mental health care at the health system level in OECD countries	Monitor changes on effectiveness and safety patients subsequent to reform of mental health services and facilitate benchmarking

Publications on indicator development, implementation, and validation within ten nations were found. Three international organizations (i.e. European Union, OECD, and WHO) developed PI for between nation comparisons. The majority of the publications (n = 90, 85%) focus on the quality of PMHC in nations where English is the native language (Australia, Canada, United Kingdom, and USA), and 66 publications (61%) are concerned with PMHC in the United States. In contrast, publications that focus on the measurement of PMHC quality in Spain, Germany, Italy, South Africa, the Netherlands, and Singapore together only account for 12% of the total sample. The majority of the publications were found in peer-reviewed journals (n = 65; 61%), the remaining publications (n = 41; 39%) consisted of reports, bulletins, and websites by governmental and non-governmental organizations. In the next sections, the performance measurement initiatives and publications per nation/region are discussed.

#### United States

In the United States, essential public (mental) health care services are jointly funded by the federal Department of Health and Human Services (DHHS) and state governments. Services are provided by state and local agencies and at a federal level administered by eleven DHHS-divisions, which include the Center for Disease Control and Prevention (CDC), the Agency for Healthcare Research and Quality (AHRQ) and the Substance Abuse and Mental Health Service Administration (SAMHSA) [1].

A considerable number of initiatives on performance measurement of the public mental healthcare in the United States at national, state, local, and service level were found. In the 1990s, the growth of managed care delivery systems in behavioral health raised the need for quality assurance and accountability instruments, and led to an increase in the number of publications on the development of performance measures in scientific literature. A total of 121 measures for various aspects and dimensions of the performance of public mental health providers, services, and systems were proposed [20,22,24,25,27,29,33,36].

In the following section, ten national initiatives that focus on between-state comparable PI are discussed in more detail. Some distinctive examples of within-state PMHC performance measurement initiatives are discussed subsequently.

One of the first, more comprehensive, and most widespread quality indicator systems in the U.S. is the *Health plan/Employer Data Information System (HEDIS)*. HEDIS is a set of standardized performance measures designed to enable purchasers and consumers to reliably compare the performance of managed care plans. Relatively few measures of mental health care and substance abuse services were included in the early versions of the HEDIS. The 2009 version only includes six measures of the performance of these services [19]. With increasing popularity of managed care plan models in PMHC, the HEDIS mental health care performance measures are widely accepted in private as well as public mental health care performance measurement projects. The measures were utilized to assess the relationship of mental health care quality with general health care quality and mental health care volume in health plans that included programs funded by state and federal governments (i.e. Medicaid) [48,64].

A set of quality indicators that is more specifically tailored to measuring the quality of mental health services was developed by the *Mental Health Statistics Improvement Program (MHSIP)*. The program aims to assess general performance, support management functions, and maximize responsiveness to service needs of mental health services and published the Consumer-Oriented Report Card including 24 indicators of Access, Appropriateness, Outcomes, and Prevention [21]. Eisen et al. evaluated the consumer surveys from both the HEDIS (the Consumer Assessment of Behavioral Health Survey; CABHS) and the MHSIP Consumer Survey. The results of this study were reviewed by several national stakeholder organizations to make recommendations for developing a survey combining the best features of each. This resulted in the development of the Experience of Care and Health Outcomes (ECHO) survey [40]. Building on the experiences with the Consumer-oriented Report Card and the advances in quality measurement and health information technology the MHSIP proposed a set of 44 PI in their *Quality Report *[71].

*The nationwide health promotion and disease prevention agenda *for the first decade of the 20^th ^century was aimed at increasing quality and years of healthy life and eliminate health disparities [31]. This agenda contained objectives and measures to improve health organized into 28 focus areas, including Mental Health and Mental disorders and Substance Abuse.

The *national association representing state mental health commissioners/directors and their agencies (NASMHPD) *provided a framework for the implementation of standardized performance measures in mental health systems [35]. A workgroup had reviewed national indicators and instruments, surveyed state mental health authorities, and conducted a feasibility study in five states. Using the MHSIP-domains as a starting point, the resulting framework includes 32 PI for state mental health systems.

The *American College of Mental Health Administration (ACMHA) *recognized the need for a national dialog, a shared vision in the field of mental health and substance abuse services, and an agreement on a core set of indicators and formed workgroup that collaborated with national accrediting organizations to propose 35 indicator definitions. These definitions were organized in three domains (i.e. access, process and outcome) applicable to quality measurement for either comparison between mental health services or internal quality improvement activities [37].

In response to the interest expressed by a number of states to develop a measure related to recovery that could be used to assess the performance of state and local mental health systems and providers, a *national research project for the development of recovery facilitating system performance indicators *was carried out. The Phase One Report on the factors that facilitate or hinder recovery from psychiatric disabilities set a conceptual framework [120]. This provided the base for a core set of system-level indicators that measure structures and processes of a recovery-facilitating environment, and generate comparable data across state and local mental health systems [68]. The second phase of the project included the development of the Recovery Oriented System Indicators (ROSI) measures based on the findings of phase one, a prototype test and review of self-report indicators in seven states, and a survey to receive feedback on administrative indicators with nine states. The ROSI consists of a 42-item consumer self-report survey, and a 23-item administrative data profile that gather data on experiences and practices that enhance or hinder recovery [76].

Parallel to the efforts to establish standardized measures of mental health and substance abuse care performance, the *National Public Health Performance Standards Program (NPHPSP) *developed three assessment instruments to assist state and local partners in assessing and improving their public health system, and guide state and local jurisdictions in evaluating their current performance against a set of optimal standards. Each of the three NPHPSP instruments is based on a framework of ten Essential Public Health Services which represent the spectrum of public health activities that should be provided in any jurisdiction. The NPHPSP is not specifically focused on the public mental health care, but it is one of the first national programs that aim to measure the performance of the overall public health system that includes public, private, and voluntary entities that contribute to public health activities within a given area [49]. Beaulieu and Schutchfield [50] assessed the face and content validity of the instrument for local public health systems and found that the standards were highly valid measures of local public health system performance. Beaulieu et al. evaluated the content and criterion validity of the local instrument, and the content validity of the state performance assessment instrument. The local and state performance instruments were found to be content valid measures of (resp.) local and state system performance. The criterion validity of a summary performance score on the local instrument could be established, but was not upheld for performance judgments on individual Essential Services [51]. After their publications in 2002, NPHPSP's public health performance assessment instruments had been applied in 30 states. The NPHPSP consorted with seven national organizations, consulted with experts in the field of public health, and conducted field tests to inform revisions of these instruments [80].

One of the first national initiatives to develop performance measures that include socioeconomic and psychosocial care focused on the development of *core performance indicators for homeless-serving programs administered by the DHHS *[52]. Based on interviews with program officials and review of existing documentation and information systems, 17 indicators that could be used by these programs were suggested, despite large differences between programs.

A pilot test of PI of access, appropriateness, outcome, and program management on a statewide basis, part of NASMHPD's S*ixteen state study on mental health performance measures*, demonstrated the potential for developing standardized measures across states and confirmed that the realization of the full potential will depend on enhancements of the data and performance measurement infrastructure. Furthermore, it was demonstrated that states can use their current performance measurement system to report comparable information [58].

An online database providing more than 300 process measures for assessment and improvement of mental health and substance abuse care was set up by the *Center for Quality Assessment and Improvement in Mental Health *(CQAIMH). Each measure is accompanied by a clinical rationale, numerator and denominator specifications, information on data sources, domain of quality, evidence basis, and developer contact information [121]. This national inventory of mental health quality measures includes many of the measures developed by the national initiatives discussed above as well as many process measures developed by individual states. It is one of the most comprehensive and broadly supported performance assessment and -improvement tools in the field of (public) mental health care to date.

In addition to the quality measurements requested by national organizations and federal agencies, some states have developed quality assessment instruments or measures tailored specifically to their data sources and mental health care system. For example, the state of Vermont's federally funded Mental health Performance Indicator Project asked members of local stakeholders in the field of mental health (i.e. providers, purchasers, and government agencies) to recommend specific PI for inclusion in a publicly available mental health report card of program performance. This multi-stakeholder advisory group proposed indicators structured in three domains, i.e. 'treatment outcomes', 'access to care', and 'practice patterns' [34].

Another example of state-specific public mental health performance measurement was found in the state of California. A Quality Improvement Committee established indicators of access and quality to provide the information needed to continuously improve the care provided in California's public mental health system. The committee adopted the performance measurement terminology used by the ACMHA and judged possible indicators against a number of criteria (such as availability of data in the California mental health system). A total of 15 indicators were formulated in four domains: structure, access, process, and outcomes. So-called special studies were designed to assess gaps in data-availability and determine benchmarks of performance [39].

Other states and localities took similar initiatives which often served a dual purpose. On the one hand, the indicators provide accountability information for federally funded programs (e.g. Minnesota, Virginia) [45,81], and on the other, the indicators provide local providers and service delivery systems with information to improve state mental health care quality (e.g. Virginia; Maryland) [53,60]. Successful implementation of such state-initiated quality assessment systems is not guaranteed. Blank et al. [61] reported on the pilot implementation of the Performance and Outcomes Measurement System (POMS) by the state of Virginia. The pilot was perceived to be costly, time-consuming and burdensome by the majority of the representatives of participating community health centers and state hospitals. Despite large investments and efforts in redesigning POMS to be more efficient and responsive, the POMS-project was cancelled due to state budget-cuts in 2002. Two years later, Virginia participated in a pilot to demonstrate the use of the ROSI survey to measure a set of mental health system PI [122].

#### Canada

Canada's health care system is publicly funded and administered on a provincial or territorial basis, within guidelines set by the federal government. The provincial and territorial governments have primary jurisdiction in planning and delivery of mental health services. The federal government collaborates with the provinces and territories to develop responsive, coordinated and efficient mental health service systems [2]. This collaboration is reflected in four publications on PMHC performance measurement discussed below.

The *Canadian Institute for Health Information (CIHI) *launched the Roadmap Initiative to build a comprehensive, national health information system and infrastructure. The Prototype indicator Report for Mental health and Addiction services was published as part of the Roadmap Initiative. The report contained indicators relevant to acute-, and community-based services whose costs were entirely or partially covered by a national, territorial or provincial health plan [83].

Adopting the indicator domains from the CIHI framework, the *Canadian Federal/Provincial/Territorial Advisory Network on Mental Health (ANMH)*, provided a resource kit of PI to facilitate accountability and evaluation of mental health services and supports. Based on literature review, and expert- and stakeholder survey, the ANMH presented 56 indicators for eight domains of performance, i.e. acceptability, accessibility, appropriateness, competence, continuity, effectiveness, efficiency, and safety [83].

Utilizing the indicators and domains from the ANMH and CIHI, the *Ontario Ministry of Health and Long-term Care (MOHLTC) *designed a mental health accountability framework that addressed the need for a multi-dimensional, system-wide framework for the public health care system, an operating manual for mental health and addiction programs, and various hospital-focused accountability tools [84].

Focusing on early psychosis treatment services, Addington et al. [85] reviewed literature and used a structured consensus-building technique to identify a set of service-level performance measures. They found 73 relevant performance measures in literature and reduced the set to 24 measures that were rated as essential by stakeholders. These disorder-specific measures cover the domains of performance originally proposed by the CIHI and utilized by the ANMH and the MOHLTC.

#### Australia

Medicare is Australia's universal health care system introduced in 1984. It is financed through progressive income tax and an income-related Medicare levy. Medicare provides access to free treatment in a public hospital, and free or subsidized treatment by medical practitioners including general practitioners and specialists. Mental health care services are primarily funded by government sources [123]. One report and one scientific publication on PI for Australian PMHC system and services were found.

The *Australian National Mental Health Working Group (NMHWG) *proposed indicators to facilitate collaborative benchmarking between public sector mental health service organizations based on the Canadian CIHI-model. Thirteen so-called Phase 1 indicators were found suitable for immediate introduction based on the available data collected by all states and territories [86].

Following major reform and ongoing deinstitutionalization of the mental health care system, Meehan et al. [87] reported on attempts to benchmark inpatient psychiatric services. They applied 25 indicators to assess performance of high secure services, rehabilitation services, and medium secure services in three rounds of benchmarking. The primary conclusion of the study was that it is possible and useful to collect and evaluate performance data for mental health services. However, information related to case mix as well as service characteristics should be included to explain the differences in service performance.

#### United Kingdom

Public mental health care in the UK is governed by the Department of Health (DH) and provided by the National Health Service (NHS) and social services. These services are paid for from taxation. The NHS is structured differently in various countries of the UK. In England, 28 strategic health authorities are responsible for the healthcare in their region. Health services are provided by 'trusts' that are directly accountable to the strategic health authorities. Eighteen publications concerning the quality of public mental healthcare in the UK were found. All but one focus on the PMHC in England, and only five studies are published in scientific peer-reviewed journals. In this section we highlight the large national initiatives.

A *National Service Framework *(NSF) for Mental Health set seven standards in five areas of PMHC (i.e. mental health promotion, primary care and access to services, effective services, caring about carers, and preventing suicide) [90]. The progress on implementation of the NSF for Mental Health was measured in several indicators per standard to assess the realization of care structures, processes, and their outcomes set out by the NSF [124].

In response to the governments' new agenda for social services, the DH issued a consultation document on a new approach to social services performance [89]. This approach included a new framework for assessing and managing social service performance that included a set of around 50 national PI for five aspects of performance: 'national priorities and strategic objectives', 'cost and efficiency', 'effectiveness of service delivery and outcomes', 'quality of services for users and carers', and 'fair access'.

The *Commission for Health Improvement *(CHI) published the first performance ratings for NHS mental health trusts [95]. These ratings were replaced by the Healthcare Commissions' framework for the NHS organizations and social service authorities [96]. The Healthcare Commission assesses the performance of mental health trusts against the national targets described in this new framework annually. In addition, the Health Commission initiated the 'Better Metrics' project aimed at providing healthcare authorities with clinically relevant measures of performance and assist local services in developing their own measures by producing criteria for good measures [98].

The *Audit Commission*, responsible for the external audit of the NHS, supported local authorities to use local PI in addition to the national services frameworks to assess their performance and responsiveness in meeting local needs by developing the local authority PI library [92]. A National Indicator Set of 188 indicators selected from this library would then become the only set of indicators on which the central government monitors the outcomes delivered by local government. The Audit Commission published the performance on these indicators annually as part of the Comprehensive Area Assessment, an effort to combine the monitoring of local services by several external auditing organizations [99].

The *Association of Public Health Observatories (APHO) *developed a series of reports to present information on the relative positions of the English Regions on major health policy areas. The mental health and drug use report contain over 70 indicators covering six areas of mental health policy: risk- protective factors and determinants; population health status; interventions; effectiveness of partnerships; services user experience; and workforce capacity [100,104].

A framework for mental health day services was developed as part of the *National Social Inclusion Program *[97]. The framework contains 34 key- and 47 supplementary indicators reflecting the different life domains and functions of day services such as community participation, mental well being, independent living and service user involvement. To provide for application in mental health services more widely, and include services such as outreach, employment and housing support services, the framework was broadened [103].

*Health Scotland *established a core set of national mental health indicators for adults in Scotland [11,125]. A set of 55 indicators was developed to provide a summary mental health profile for Scotland, enable monitoring of changes in Scotland's mental health, inform decision making about priorities for action and resource allocation, and enable comparison between population groups and geographical areas.

#### Non-English speaking nations

Ten scientific publications in peer-reviewed journals and one report by a governmental organization concerned with the quality of PMHC in non-English speaking countries were included. These studies and initiatives are discussed briefly in this section.

*Gispert *et al. [105] calculated the mental health expectancy of the population and a Spanish region to show the feasibility of a generic mental health index which covers both duration of life and a dimension of quality of life.

In Germany, an expert group consisting of professionals, patients, and policy makers from state mental hospitals, psychiatric departments, and health administrations defined 23 quality standards, for 28 areas of inpatient care, at three levels of quality assessment in psychiatric care [106]. *Bramesfeld *et al. [107] applied a concept of responsiveness developed by the WHO to evaluate German inpatient and outpatient mental health care. They conclude that responsiveness as a parameter of health system performance provides a structured way to evaluate mental health services. However, the instrument proposed by the WHO to assess responsiveness was found to be too complicated and in-depth for routine use in guiding improvement in mental health care.

In The Netherlands, *Nabitz *et al. [109] applied a concept-mapping strategy to develop a quality framework for addiction treatment programs. Nine clusters on two dimensions were identified. The three most important clusters were named 'attitude of staff', 'client orientation' and 'treatment practice'. *Roeg *et al. [108] applied a similar concept-mapping strategy with Dutch experts to develop a conceptual framework for assertive outreach programs for substance abusers and formulated nine aspects of quality as well. They classified these aspects in structure, process and outcome, and found the clusters named 'service providers' activities', 'optimal care for client' and 'preconditions for care' to be the most important aspects of care in relation to quality.

An assessment of the validity of 11 PI for the Dutch occupational rehabilitation of employees with mental health problems showed evidence on the content validity of these PI, but could not establish a relation between these PI and outcome [110]. Indicators of pre- and post admission care were applied to assess the quality of another modality of Dutch public mental health care, i.e. compulsory mental health treatment, to conclude that these indicators are useful measures of mental health care utilization [111].

The only governmental report on PMHC performance measurement in non-English speaking nations around the world was published by the *Dutch Health Care Inspection's Steering Committee-Transparency Mental Healthcare *which presented a basic set of 32 PI for assessment of effectiveness, safety and client-centeredness of mental health care, addiction care, and forensic care services to provide the public with quantifiable and understandable measures for the quality of care [112].

A team of Italian researchers derived 15 indicators of guideline conformance from several schizophrenia treatment guidelines [113]. They found these PI to be a simple and useful tool to monitor the appropriateness of schizophrenia treatment provided by public institutions.

To assess the balance of resource allocation between community and hospital-based services in South Africa, *Lund and Flisher *developed indicators measuring staff distribution and patient service utilization [114]. They conclude that community/hospital indicators provide a useful tool for monitoring patterns of service development over time, while highlighting resource and distribution problems between provinces.

Finally, in Singapore two psychiatrists identified 13 process indicators from literature and guidelines that assess the quality of an early psychosis treatment intervention to inform clinicians on their treatment and to provide a tool for policymakers [115].

#### International

Next to these national/regional/local indicator sets and quality measurement frameworks, three international organizations, i.e. the European Commission (EC), the Organization for Economic Cooperation and Development (OECD), and the World Health Organization (WHO) reported on their efforts to develop PI for standardized quality measurement and comparison of PMHC quality between nations.

The *Commission of the European Communities' National Research and Development Centre for Welfare and Health (STAKES) *has coordinated a project to establish a set of indicators to monitor mental health in Europe. The proposed set contained 36 indicators covering health status, determinants of health, and health systems, based on meetings with representatives of mental health organizations in the member states and other organizations including WHO-Euro, OECD, EMCDDA and Eurostat. Validity, reliability and comparability of the drafted set of indicators were further tested by collecting data from existing data sources and conducting a pilot survey. Only some of the data collected were found to be reliably comparable and available as national mental health systems differed substantially in organization and structure [8].

The *Organization for Economic Cooperation and Development (OECD) Health Care Quality Indicators Project *identified priority areas for development of comparable indicators for the technical quality of national health systems, using a structured review process to obtain consensus in a panel of experts and stakeholders from 21 countries, the World Health Organization (WHO), the World Bank, and leading research organizations. 12 indicators were proposed covering treatment, continuity of care, coordination of care, and patient outcomes [10]. As in the EU--STAKES project, OECD researchers found that selecting a set of indicators for international use is constrained by the limited range of data potentially available on a comparable basis in many countries.

To assess key components of a mental health system and provide essential information to strengthen mental health systems, the *WHO developed the Assessment Instrument for Mental Health Systems (WHO-AIMS 2.2)*. The 10 recommendations for mental health system development published in the World Health Report 2001 served as the foundation for the WHO-AIMS. Expert and stakeholder consultation, pilot testing in 12 resource-poor countries and a meeting of country representatives, resulted in an instrument consisting of six domains and 156 items which were rated to be meaningful, feasible, and actionable. As the six domains are interdependent, conceptually interlinked, and overlapping, all domains need to be assessed to form a relatively complete picture of a mental health system [116]. Thus, the WHO-AIMS can be viewed as a multi-item scale, in contrast to indicators proposed in the EU and OECD programs which were focused on single-item indicators.

### Characteristics of PMHC performance indicators

A total of 1480 unique PI are included in the inventory. 370 indicators of these are represented in two or more publications. To assess individual PI we focused on characteristics reported by the developers in terms of method of development, level of assessment, Donabedian's domain of care, dimensions of performance, diagnosis or condition, and data source, as presented in Table 2.

**Table 2:** Classifying unique performance indicators for public mental health care

Indicator characteristic		Descriptive statistics	
		
		n	%
Development method			
	Expert opinion	401	27.1
	Structured consensus method	177	12.0
	Literature review/application of pre-existing instruments	239	16.1
	Mixed literature and stakeholder consultation	604	40.8
	Method not specified	59	4.0
Level of assessment			
	Clinician	7	0.5
	Service	650	43.9
	System/Health plan	823	55.7
Care domain			
	Structure	258	17.4
	Process	690	46.6
	Outcome	532	35.9
Dimensions of performance			
	Effectiveness/Improving health/Clinical focus	633	42.8
	Accessibility/Equity	289	19.5
	Responsiveness/Patient focus/Acceptability	136	9.2
	Competence/Capability	104	7.0
	Efficiency/Expenditure/Cost	42	2.9
	Safety	55	3.7
	Appropriateness	152	10.3
	Continuity/Coordination	63	4.3
Diagnosis or condition			
	Homelessness	33	2.2
	Substance abuse disorder	121	8.2
	Mood disorder	94	6.4
	Psychosis/schizophrenia	124	8.4
	Other diagnosis/condition specific	74	5.0
	Across disorders/populations	1034	69.9
Data source			
	Survey/Audit	419	28.2
	Administrative data/Medical record	607	41.0
	Multiple sources	121	8.2
	Not specified	333	22.5

More than a quarter of the PI are based solely on expert opinion and more than half of the PI are developed using both literature review and expert consultation, with 12% utilizing a structured consensus procedure such as adaptation of the RAND-method or a modified Delphi procedure [e.g. [21,79,82]]. For 59 of the included PI no method of development was specified in the publication.

With regard to the level of assessment, the majority of the PI (55.7%) aim to assess the quality of a PMHC system. These PI incorporate data from multiple service providers within a region (e.g. county, state, province, nation) to measure the standard of PMHC-quality either against benchmarks set by the regional legislator, or against the PMHC-quality in other regions. Only 7 PI were specifically designed to assess the performance of individual clinicians, i.e. physicians in a community mental health center [24]. The remaining PI (43.9%) measure the performance on a service-level to improve quality of care, gain transparency for purchasers, or inform patient-choice.

We found more than a third of the PI measure performance in terms of treatment outcome, for instance in suicide rates, crime rates, or incidence rates of homelessness. Almost half of the included PI are process measures. Based on guidelines that specify 'best practice' treatment processes, for instance in terms of duration, contact intensity, or medication dosage, these PI are usually formulated as a proportion of a population that is provided with a treatment according to guidelines. However, there are differences in both the guidelines used (e.g. the number of days between discharge from inpatient treatment and the first outpatient contact varies between 1 and 30), and in the population used in the denominator of the PI (e.g. the percentage of the population in a region vs. the population that receives treatment).

Dimension of performance is the most diverse categorization of PI. To structure the PI from different regions and developers by dimension of performance, some concepts that are strongly related were grouped, resulting in eight main dimensions of performance. 42.8% of the PI aim to measure the 'effectiveness' of PMHC and provide stakeholders with a measure of degree of achieving desirable outcomes. Another 19.5% of the PI are designed to measure the 'accessibility' and 'equity' of PMHC. Remarkably, only 2.9% of the PI assess the efficiency, cost, or expenditure of PMHC.

A large majority of the PI (69.9%) is not specific to any diagnostic group or a group with a certain condition (such as homelessness). Relatively many of the diagnosis-specific PI are developed for psychosis-related disorders (8.4%) and substance abuse disorders (8.2%), as many individuals in the target-group of PMHC cope with these disorders.

Finally, the data source of the included PI was inventoried. For a considerable number of PI (22.5%), no data source was specified. Development of these PI did often include the specification of the data needed for the PI, but did not identify a data source. More than half of the PI for which a data source was specified are based on administrative data or medical records.

### Feasibility, data reliability and validity of PMHC performance indicators

Aspects of feasibility and content validity of PI can be established through literature review and expert consultation. Almost all PI development initiatives have used literature review and/or expert-consultation methods to establish at least some evidence on the content validity of the proposed PI. Furthermore, through stakeholder-consultation techniques, ranging from telephone interviews and expert meetings to structured consensus procedures, possibilities for implementation and support for the PI in the field is often assessed as well, thus establishing evidence for the feasibility of the PI. With regard to content validity and feasibility (i.e. support in the field and expected reliability) we confine ourselves to remarking that for all the PI included in this inventory, some evidence for the content validity and feasibility has been established. However, the strength of the evidence varies and depends heavily on the methods used to consult stakeholders and experts [126].

Although forms of reliability that are relevant to PI based on surveys and audits, such as test-retest reliability and inter-rater reliability, have been examined by several indicator developers [e.g. [71,76,88]], reliability in terms of accuracy and completeness of the (administrative) data sources used for PI has rarely been assessed. A number of organizations and authors have recognized that information contained in databases and patient records may be incomplete, inaccurate, or misleading which can have a considerable effect on the usability and feasibility of the PI [e.g. [21,52,69]]. However, we found only two studies that assessed the accuracy or availability of the information needed for the PI. Huff used data from the Massachusetts state contractor's claims data set used for paying providers and data from the Medicaid beneficiary eligibility file. The author considered key fields in the claims dataset to be of high reliability because those data elements are essential in determining service reimbursement and the timeline of the payments. The eligibility file had some reliability problems with a small subset of duplicate cases and missing values in specific fields, in particular 'beneficiary race'. Huff concluded that both data-files were sufficiently reliable, at least for the intended study [32]. Garcia Armesto et al. provided an overview of mental health care information systems in 18 OECD countries to support the implementation of the OECD system level PI selected in 2004. They conducted a survey in each of the participating countries to gather information on the types of system-level mental health data available, the data sources available on a national level, and the institutional arrangements on ownership and use of the information systems and concluded that data on mental health care structures and activities is generally available but data necessary for measurement of mental health processes and outcomes is more problematic. Furthermore, the integration of information systems across different levels of care provision (i.e. inpatient, outpatient, ambulatory, and community care) was found to be low [119]. The mixed results presented by these two studies show that data reliability (accuracy as well as completeness) cannot be presumed to be sufficient for the implementation of PI for PMHC.

We found 18 publications that focused on the relation between an indicator and an external criterion. A broad range of criteria have been used in these assessments of the criterion and construct validity of PI. The criteria vary in perspective (covering subjective quality or technical quality), domain of care (measures of structure, process or outcome), and in data source (questionnaires, audits, or administrative data). The studied PI, the criteria used to validate the PI, and the outcome of the study are shown in Table 3.

**Table 3:** Published descriptions and properties of unique performance indicators for public mental health care

Study	Performance indicator	Related criterion	Result
Simpson & Lloyd [17]	• Methadone maintenance (MM) drug abuse treatment client evaluation score (composite measure from 7 items) • Therapeutic communities (TC) drug abuse treatment client evaluation score (composite measure from 7 items) • Out-patient drug-free (DF) drug abuse treatment client evaluation score (composite measure from 7 items) • outpatient detoxification (DT) drug abuse treatment client evaluation score (composite measure from 7 items)	1 year post treatment (high scores more favorable): • opoid use • nonopoid use • marijuana use • alcohol use • employment • jail • return to treatment within 1 year • composite score (all above mentioned criteria)	• More positive MM treatment client evaluation was sig. pos. related to 1-year post treatment opoid use, nonopoiduse, return to treatment, and the composite score. • More positive TC treatment client evaluation was sig. pos. related to 1-year post treatment opoid use, nonopoiduse, marijuana use, employment, jail, and the composite score. • More positive DF treatment client evaluation was sig. pos. related to 1-year post treatment opoid use, nonopoiduse, marijuana use, and the composite score. • More positive DT treatment client evaluation was sig. pos. related to 1-year post treatment return to treatment

Srebnik et al. [22]	• Satisfaction domain: client satisfaction questionnaire; involvement in treatment; treatment appropriateness; safety at mental health center • Functioning domain: physical; mental; social and leisure; skills for handling stress and symptoms • Quality of life domain: safety; concerns about living condition; goal attainment; victimization • Clinical status domain: four-dimensional classification scale	• Percentage of clients with any meaningful activity • Percentage of clients with a independent living situation • Percentage of clients with no out-of-community (hospital/jail admission) episode	• No sig. associations of satisfaction measures with any of the criteria • Sig. pos. association of functioning measures with living situation • Sig. neg. association of functioning measures with out-of-community episode • Sig. pos. association of quality of life measures with meaningful activity • Sig. pos. association of clinical status measure with meaningful activity, living situation, and out-of-community episode

Druss et al. [30]	• Promptness and continuity of outpatient follow-up after discharge • Any outpatient follow-up after discharge • Length of stay • Readmission within 30 days • Readmission within in 180 days and total days readmitted within 180 days	Individual level and hospital level measures of satisfaction with: • General service delivery • Alliance with inpatient staff	• Sig. pos. association of promptness and continuity of outpatient follow-up and alliance with inpatient staff at individual level and at hospital level • Sig. pos. association of any outpatient follow-up and alliance with inpatient staff at individual level • Sig. pos. association of length of stay and alliance with inpatient staff at individual level • No sig. association of early readmission with any of the criteria • Sig. neg. association of readmission intensity and general service delivery at individual level

Macias et al. [28]	• International Center for Clubhouse Development Certification status	• 3 organizational resource variables • 7 survey variables reflective of clubhouse model fidelity	• No sig. association of resource variables and clubhouse certification status • Sig. pos. association with 6 of the 7 fidelity variables.

Huff [32]	• Crisis service utilization within 30 days after discharge • Median index episode length of stay • Median number of service contacts within 30 days after discharge • Number of providers contact within 30 days after discharge • Diagnostic evaluation services within 30 days after discharge • Early ambulatory contact (within 5 days) • Medication management service within 30 days after discharge • Psychotherapy service within 30 days after discharge	• Readmission for an acute episode of care to any acute mental health provider within a 30-day period after being discharged	• Sig. pos. association of crisis service utilization and 30-day acute relapse risk • No sig. association of length of stay and 30-day acute relapse risk • Sig. pos. association of service contacts and 30-day acute relapse risk • Sig. pos. association of provider contacts and 30-day acute relapse risk • Sig. neg. association of diagnostic evaluation services and 30-day acute relapse risk • Sig. neg. association early ambulatory contact and 30-day acute relapse risk • Sig. neg. association of medication management services and 30-day acute relapse risk • Sig. neg. association of psychotherapy service and 30-day acute relapse risk

Shipley et al. [91]	• Mean patient satisfaction score (4-item questionnaire) • Mean clinician satisfaction score (4-item questionnaire) • Mean referrer satisfaction score (4-item questionnaire) • Mean time form referral to first appointment • Proportion of patients in which referrer is notified of contact within 6 weeks of referral • Proportion of patient referred and offered appointments who attended	• Five clinical teams of a psychiatric service, one of which was regarded as seriously deficient by an independent review. All teams had important differences in type of referrals and diagnostic group	• Sig. differences in patient satisfaction between teams with which the poorly performing team could be identified • No sig. differences in clinician satisfaction between teams • No sig. differences in referrer satisfaction between teams • Sig. differences in time to first appointment PI that failed to identify deficient team. • Sig. differences referrer notification that failed to identify deficient team. • No sig. differences of patient attending appointments PI between teams.

Chinman et al. [41]	• Service-need index, incorporating ratings of drug and alcohol use, patient's average acuity score, and GAF-m score	• Average caseload of treatment team or program. • Average number of outpatient service hours provided to patients	• Sig. neg. correlation between service-need index and caseload • Sig. pos. correlation between service-need index and service hours provided

Dausey et al. [44]	• Preadmission care (binary variable, continuous variable, spline variable)	• Duration of index admission (length of stay). • Use of post discharge aftercare at 30 days. • Readmission at 14, 30 and 180 days.	• Sig. neg. association between preadmission care and length of stay. • Sig. pos. association between preadmission care and aftercare at 30 days • Preadmission care is associated with a slight increase in probability of readmission

Davis & Lowell [42,43]	• Percentage of funds allocated to state hospitals to community-based services is at the theoretical optimum proportions (43% to 57%)	• Suicide rate per 100,000 population • Cost per capita for mental health care	• Suicide rate lower in states in which funds allocation proportion are close to the optimum. • Relation between expenditure and cost per capita is only found when states that differ more than 12% from the ideal funding partition are excluded

Beaulieu et al. [50]	• NPHPSP Local public health system performance measurement instrument	• Documentary evidence • External judge rating of performance	• Documentation to support agencies' responses to the local instrument validated their responses • External judge ratings were unreliable due to lack of knowledge of local systems

Edlund et al. [59]	• Percentage of persons with any alcohol, drug, or mental disorder that received at least 4 visits with a mental health specialist or 4 visits with a primary care provider that included counseling for mental health problems and/or that received medication that was efficacious for the individual's disorder and used at a dosage exceeding the minimum recommended dosage for an adequate duration • Active treatment after assessment: use of inpatient, day treatment, or residential care; use of prescribed psychotropic medications daily for a month or more; or a period of potentially therapeutic outpatient treatment for alcohol, drug or mental conditions	• Overall satisfaction with the mental health care available for personal or emotional problems during the past 12 months	• Sig. pos. association of appropriate counseling/appropriate pharmacotherapy, and satisfaction with available mental health services • Sig. pos. association of active treatment, and satisfaction with available mental health services

Charbonneau et al. [62]	• Dosage adequacy: antidepressant average daily dosage during 3-month profiling period meets guideline-recommended minimum daily dosage • Duration adequacy: inadequate duration defined as > 21% of the profiling period without antidepressants • Follow-up visit adequacy: at least 3 visits to primary care or psychiatry clinics within 3 months of the initial depression encounter; at least 2 visits in addition to the initial one within 3 months of diagnosis	• Inpatient overall, and psychiatric hospitalizations during the 12 months after the depression care period	• No sig. association between dosage adequacy and any criteria • Sig. neg. association between duration adequacy and subsequent overall or psychiatric hospitalizations • No sig. association between follow-up visit adequacy and any criteria

Druss et al. [64]	• Medication during at least 3 follow-up mental health care visits in the 3 months after a new depressive episode • Ongoing medication treatment in the 3-month period after a new depressive episode • Ongoing medication treatment in the 6 months after a new depressive episode • Percentage of members hospitalized for a mental disorder who had an ambulatory visit with a mental health care provider within 30 days of hospital discharge • Percentage of members hospitalized for a mental disorder who had an ambulatory visit with a mental health care provider within 7 days of hospital discharge	• Volume of ambulatory mental health use • Volume of inpatient mental health discharges Volume of inpatient mental health days.	• Sig. pos. association between volume of ambulatory mental health use, and medication management PI, and outpatient follow-up PI • Sig. pos. association of volume of inpatient mental health discharges, and medication management PI, and outpatient follow-up PI • Sig. pos. association of volume of inpatient mental health, and medication management PI, and outpatient follow-up PI

Leff et al. [66]	• Service fit: the congruence between services prescribed or needed and services received	• Mortality: natural deaths, medico-legal deaths, suicides	• The relationship between service fit and mortality is more apparent in models based on medico-legal deaths and suicides than in the model based on natural deaths

Nieuwen-huijsen et al. [110]	• Assessment of symptoms (2 criteria), one of both criteria not met within 2 consultations. • Correct diagnosis (3 criteria), one of more criteria not met within 2 consultations. • Evaluation curative care (2 criteria), one of both criteria not met within 2 consultations. • Assessment of work-related causes (2 criteria), one of both criteria not met within 2 consultations. • Evaluation of work disabilities (2 criteria), one of both criteria not met within 2 consultations. • Interventions targeted at the individual (1 criterion), criterion not met within 3 consultations. • Interventions targeted at organization (1 criterion), criterion not met within 3 consultations. • Interventions targeted at providers of care in curative sector (2 criteria), one or both criteria not met within 3 consultations. • Advice on return to work (2 criteria), one or both criteria not met at each consultation. • Timing of consultations (2 criteria), criterion 1 not met at first consultation or criterion 2 not met at consultation 2 or 3. • Summed score over 9 indicators with sufficient content validity and variability.	• Time to return to work • Change in level of fatigue • Patient satisfaction	• No sig. association assessment of symptoms and any criteria • No sig. association correct diagnosis and any criteria • Sig. pos. association evaluation curative care and satisfaction. • No sig. association assessment of work-related causes and any criteria • Sig. neg. association evaluation work disabilities and return to work. • No sig. association organizational interventions and any criteria • Sig. pos. association interventions curative sector and return to work. • No sig. association advice to return to work and any criteria • Sig. neg. association timing of consultations and return to work. • Sig. pos. association overall quality of care and return to work, and satisfaction.

Rost et al. [70]	• A prescription for an antidepressant medication was noted from up to 30 days before to 14 days after index episode start date; dosage sufficient to take medication for 84 out of 114 days following first prescription; 3 non-emergency visits room visits to a primary care or mental health provider at least one of them had to be with the prescribing provider • 4 or more specialty depression care counseling visits in the 6 months following the index visit	• Absenteeism: lost work hours in the past 4 weeks due to illness or doctor visits	• No sig. association appropriate medication and change in absenteeism over 1 year • Sig. association appropriate psychotherapy and change in absenteeism over 1 year

Wierdsma et al. [111]	• Clients receive any psychiatric care in the year before involuntary admission	• Length of stay (less than 3 weeks; more than 6 months; mean number of days) • Ambulatory follow-up • Readmission (within 3 months; within 1 year) • Continued care 12 months after involuntary admission	• Sig. pos. association preadmission care and length of stay, and continued care after 12 months. • No sig. association preadmission care and ambulatory follow-up, and readmission

Six studies have focused on the relations between measures of (client) satisfaction and indicators of technical PMHC quality. Either by assessing the relation of a measure of satisfaction with an external criterion [17,22] or using measures of satisfaction as criteria for PMHC quality to study the usefulness of PI of PMHC processes and (clinical) outcomes [30,59,91,110]. Four of these studies show significant associations between the satisfaction measure with measures of effectiveness, appropriateness, accessibility, and responsiveness. Another study reported relations between measures of satisfaction and measures of effectiveness and appropriateness as well, but those associations disappeared when client-level data were aggregated to reflect the quality on a service level of assessment. A relation between satisfaction and a measure of continuity of care, however, did remain significant on both levels of assessment. One study did not find any association of measures of client satisfaction with community-valued outcome indicators, such as involvement in meaningful activities or residential independence. Thus, we found evidence to support the criterion validity of satisfaction measures, specifically as measures of the continuity of PMHC. However, measures of client satisfaction seem to be less useful in the assessment of the long-term effect of PMHC on a population in its catchment area.

Two studies assessed the validity of preadmission care as an indicator for the quality of public mental health services [44,111]. Both studies found a relation between preadmission care and post-discharge use of care. The associations of preadmission care measures and measures of readmission found in these studies were more mixed. Wierdsma et al. found no relations between preadmission care and readmission within 90 days or within one year, and Dausey et al. found that clients who received preadmission care were slightly more likely to be readmitted within 14, 30 and 180 days after discharge. The studies show contradictory results on the associations of preadmission care and the length of stay. In one study length of stay is increased when the clients receives any care before admission, while the other study reports a decrease in length of stay when any preadmission care is received. Based on the results of these studies, receiving preadmission care can be considered to be useful in assessment of the continuity of PMHC. However, the validity of 'length of stay' as a criterion for PMHC quality is questionable. A study by Huff used readmission within 30 days after discharge for an acute mental health care need as a criterion measure and showed no association of median length of stay and this criterion [32]. Length of stay was the only measure of the eight measures assessed in this study that showed no relation with the criterion.

PI for the appropriateness of depression care, and their relation to mental health care outcomes and structures were assessed in three studies [62,64,70]. The PI assess appropriateness expressed as the ratio of clients receiving outpatient depression care, which receive guideline-conformant medication dosage, medication duration, and follow-up visits. The results of these studies with regard to the criterion validity of these PI vary. The two studies that assessed the relation of dosage adequacy did not find an association with the outcome criteria. Two studies that assessed (measures of) appropriate medication duration did find associations with volume of care, and post-care period hospitalizations, but the one study that included medication duration in an indicator of appropriate medication did not find a relation with the outcome-criterion absenteeism. The results on measures of follow-up visit adequacy were mixed as well. Associations with volume and absenteeism were shown, but no relation to post-care period hospitalizations was found.

Two studies proposed PI that included aspects of service need and assessed their usefulness [41,66]. In showing that these PI are associated with PMHC processes (i.e. average caseload and provided service hours) and outcomes (i.e. medico-legal and suicide mortality rates), these studies contributed to the evidence on the usefulness of PI that incorporate service-need of clients.

The validity of PI developed by (semi-) governmental organizations was assessed in only two studies published in peer-reviewed journals. Druss et al. used HEDIS measures of medication management and follow-up to assess the volume-quality relationship and found significant associations between both follow-up and medication management measures, and volume of mental health services [64]. A study by Beaulieu et al. assessed the criterion validity of the performance measurement instrument for local public health systems, developed by the CDC [50]. They reported on the association of responses on the instrument and documentary evidence and found that it validated the responses. However, a second method employed to validate the response on the instrument against ratings by external judges proved to be unreliable due to lack of knowledge of the local systems of the judges.

Three studies assessed the validity of measures of mental health care structure as measures of quality. Macias et al. assessed the potential worth of model-specific mental health program certification as a core component of state and regional performance contracting with mental health agencies. Based on an evaluation of International Center for Clubhouse Development prototype certification program, they conclude that a model-based certification program can attain sufficient validity to justify its inclusion in mental health service performance contracting [28]. Davis and Lowell suggested an optimum ratio of state-operated to community-operated psychiatric hospital beds and assessed the relation of (deviation from) this ratio to suicide rate, and cost of mental health care. The results of these studies show that suicide rate is lower in states in which these ratios were close to the theoretical optimum. The relationship of the optimum ratio and cost per capita was less clear cut. A linear relationship was found only when outliers were excluded [42,43].

Finally, Nieuwenhuijsen et al. assessed the relationship of ten process measures and their summed score with two measures of outcome i.e. time to return to work, and change in level of fatigue. Time to return to work was found to be related to only three of the process measures and the summed score, and no significant relations between change in level of fatigue and any of the process measures were found. Thus, although content validity of ten of the eleven PI was established, the criterion validity of most of the separate PI was not [110].

## Discussion

This systematic review set to provide insight into the state of quality assessment efforts for public mental health care (PMHC) services and systems around the world, the characteristics of performance indicators (PI) proposed by these projects, and the evidence on feasibility, data reliability and validity of PI for PMHC.

The systematic inventory of literature resulted in the inclusion of 106 publications that specified PI, sets of PI, or performance frameworks for the development of PI. 1480 unique PI for PMHC were proposed covering a wide variety of care domains and quality dimensions. Establishment of aspects of feasibility and content validity of PI seem to be an integral part in indicator development processes. Through review of literature, expert consultation, or stakeholder consensus almost all publications show that the PI under development can be implemented, and measure a meaningful aspect of health care quality. We found that for almost a quarter of the PI no data source was specified in the publication. Most of the remaining PI (53%) are based on administrative data. Eighteen publications, 17% of the total, reported on the assessment of criterion validity of PI for PMHC. In these publications, the criterion validity of 56 PI was assessed, less than 4% of the total. This percentage is even lower when we take into account that several studies assessed similar PI.

The majority of the publications focused on PMHC systems and services in the United States and over 80% of the publications were concerned with PMHC systems in English-speaking nations. This could be explained by the organizational structure of the U.S. health care provision and payment system, which is primarily operated by private sector organizations, has traditionally put a relatively large emphasis on transparency and accountability of costs and performance of health care providers. The introduction of managed care techniques and organizations in U.S. mental health care in the late 90's has spurred the development of quality assurance instruments even further. This resulted in a plethora of PI to provide local, state, and federal administrators with information for PMHC policy and -funding purposes as well as to guide quality improvement efforts. The skewness of the distribution of publications towards PMHC in English-speaking nations is possibly exaggerated by including only English and Dutch publications in the review. As performance measurement programs and efforts are predominantly focused on PMHC within a nation, they are likely to be published in the language of that nation. However, the structure of the healthcare system may have a profound effect on the efforts put into performance indicator research.

More than 40% of the PI aims to measure the effectiveness or clinical focus of PMHC However, the remaining PI measure a wide variety of performance dimensions. This could indicate a lack of consensus on the definition of PMHC quality between nations and even within nations. The diversity of performance dimensions in PI is also indicative of (local) political interests in PMHC. When designing PI for PMHC systems or services, developers often consider the local political climate and interests, particularly as the policymakers and politicians are the main stakeholders and primary users of the PI.

Only a relatively small number of PI combine data from multiple sources. Although the PI aim to measure performance on a system level of care, data systems of service providers are probably still 'stand-alone'. Issues such as privacy, absence of unique identifiers, data ownership, and lack of standard data formats could prevent data systems from integrating at the same rate as the service provision.

The hazards and risks of inadequate data reliability in terms of completeness and accuracy, for the usability and feasibility of PI based on administrative data sources, have been recognized by a number of authors and leading organizations in the field of performance measurement [e.g. [71,119]]. It is therefore surprising we only found two publications that explicitly assessed the reliability of administrative databases for PI in PMHC. It seems developers assume data reliability, at least availability and completeness, based on expert opinion and stakeholder consultation. However, providers collecting the data often have interests in the conclusions drawn from PI and when they're asked by external organizations to extract data from their client-registration systems, data reliability cannot be assumed. Especially when services or systems benefit from better performance, or the purpose of the PI is unclear to the unit (i.e. person or department) responsible for collecting the data, data reliability should be evaluated.

The consultation of experts and stakeholders not only proves to be a widely accepted method to ensure face validity and contribute to the content validity of PI, but seems an important tool to create support in the field to use the PI for accountability and transparency purposes by (external) accrediting organizations and PMHC financing bodies, or (internal) quality monitoring and improvement by PMHC care providers as well.

For only a fraction of the 1480 unique PI included in this inventory the relationship with criteria of quality has been assessed. An explanation for this finding is that criterion validity research is time-consuming and costly, and the added value is not always apparent to stakeholders. The performance on both the indicator and the criterion of a sufficiently large research group that is representative for the client population needs to be recorded in order to reliably assess the extent of the correspondence between indicator and criterion. When consensus between stakeholders on the usefulness and feasibility of PI has been procured, indicator developing organizations often do not have the funds or the incentives to further study the validity of the PI and prioritize the utilization of the PI to increase transparency or accountability of the PMHC system. Understandably, these stakeholders have more interest in the information generated with PI than in 'fundamental' characteristics of PI themselves, such as criterion validity.

While the majority of the associations between the PI and the criteria studied in the included publications are statistically significant and in the expected direction, studies report mixed and in some cases even contradictory results in several PI. Measures of satisfaction, readmission, certification status, medication dosage adequacy, length of stay, and appropriateness of screening are reported to have no significant association with one or more criteria of PMHC quality. However, other studies do report significant associations of some of the same measures with other criteria, or even use these measures as criteria to validate others. The scientific and practical utility of criterion validation depends as much on the measurement of the criterion as it does on the validity of the indicator [15]. For many concepts related to PMHC quality, valid criteria are simply not available.

## Conclusions

The pool of indicators that have been developed assess the quality of public mental health care systems is remarkable in both size and diversity. In contrast, very little is known on several elementary psychometric properties of PI and the construct of quality of public mental health care.

Efforts should be made to solve issues with regard to data system integration, as they limit the applicability of PI, specifically on a system- or population level of measurement. Furthermore, an assessment of the information-infrastructure can be highly beneficial for the usefulness and feasibility of newly developed PI and should be an integral part of indicator development initiatives.

Demarcation of the construct of PMHC quality and definition of meaningful criteria against which PI can be validated should be the focus of future research. As the need for, and use of PI in PMHC increases, assuring the validity of PI becomes a priority to further the transparency, accountability and quality improvement agenda of PMHC.

## Competing interests

The authors declare that they have no competing interests.

## Authors' contributions

SL carried out the literature search, selection of publications and classification of performance indicators, participated in the design of the study, and drafted the manuscript. MCAB participated in selection of publications and classification of performance indicators, participated in the design of the study, and helped to draft the manuscript. MASDW participated in selection of publications and classification of performance indicators, participated in the design of the study, and helped to draft the manuscript. OAA participated in the design of the study and helped to draft the manuscript. NSK participated in the design and coordination of the study and helped to draft the manuscript. All authors read and approved the final manuscript.

## Pre-publication history

The pre-publication history for this paper can be accessed here:

http://www.biomedcentral.com/1471-2458/12/214/prepub
